# Microbiological and Mycotoxicological Quality of Common Wheat in Romania in the Extremely Dry 2023–2024 Agricultural Year

**DOI:** 10.3390/toxins17040154

**Published:** 2025-03-22

**Authors:** Valeria Gagiu, Elena Mirela Cucu, Alina Alexandra Dobre, Gina Pusa Pirvu, Oana Alexandra Oprea, Cristian Mihai Pomohaci, Elena Mateescu, Nastasia Belc, Doru Ioan Marin

**Affiliations:** 1 National Research & Development Institute for Food Bioresources (IBA Bucharest), 020323 Bucharest, Romania; valeria.gagiu@bioresurse.ro (V.G.); alina.dobre@bioresurse.ro (A.A.D.); gina.constantinescu@bioresurse.ro (G.P.P.); cristian.pomohaci@fifim.ro (C.M.P.); nastasia.belc@bioresurse.ro (N.B.); 2 Faculty of Agriculture, University of Agronomical Sciences and Veterinary Medicine Bucharest (USAMV Bucharest), 011464 Bucharest, Romania; doru.marin@agro-bucuresti.ro; 3 National Meteorological Administration (METEO-Romania), 013686 Bucharest, Romania; elena.mateescu@meteoromania.ro; 4 Faculty of Land Improvement and Environmental Engineering, University of Agronomical Sciences and Veterinary Medicine Bucharest (USAMV Bucharest), 011464 Bucharest, Romania

**Keywords:** wheat quality, water activity, *Fusarium*-damaged kernels, deoxynivalenol, aflatoxin, carcinogenic risk, heatwave, extreme drought, atmospheric systems (heat dome and omega block), climate change

## Abstract

This study examines the microbiological and mycotoxicological quality of common wheat in Romania in the extremely dry 2023–2024 agricultural year. Common wheat grown in the West Plain, Southern Hilly Area, Transylvania, and northern Moldavia (45–48° N, 21–27° E) had higher moisture content, water activity, *Fusarium*-damaged kernels, and deoxynivalenol levels. This was due to moderate temperatures, abundant precipitation, and soil water reserves in May, followed by moderate drought from June to August. Conversely, common wheat from the Oltenia Plain, the Southern Plain, and southern Moldavia (43–46° N, 23–28° E) had the lowest contamination levels, attributed to extreme temperatures and drought during June–August. Common wheat from Dobrogea (45° N, 28° E) showed the highest total fungi contamination, which was influenced by precipitation at harvest. Although microbiological and mycotoxicological contamination was low, it negatively affected the physico-chemical and sensory–colorimetric parameters of common wheat, particularly in the West Plain, Oltenia Plain, and Dobrogea. Consequently, there could be significant economic losses for farmers, storekeepers, millers, and bakers, as well as a decline in the quality of finished foods. Moreover, the coexistence of deoxynivalenol and total aflatoxins in common wheat grown in the northwest of the country indicates the spread of contamination due to dry conditions and climate change.

## 1. Introduction

Common wheat (*Triticum aestivum*) is cultivated in temperate regions and plays a crucial role in the economy of many countries and global food security [1,2,3]. Climate changes until 2100 will harm cultivated areas, productivity and the quality of wheat harvests in warm regions, and positively affect colder regions [1,4]. Also, climate changes have increased the incidence and level of contamination with fungi and their mycotoxins (*Fusarium* spp., deoxynivalenol; *Aspergillus* spp. and *Penicillium* spp., aflatoxins) in cereals; as a result, the health risks associated with the consumption of contaminated grains and food will increase [5,6,7,8,9,10,11,12,13,14].

The year 2024 was the warmest since 1850, with an average temperature of 15.10 °C globally and 10.69 °C in Europe [15,16]. The extreme temperatures in the summer of 2024 were determined by a combination of factors such as global warming and the presence of the El Niño phenomenon in the tropical Pacific Ocean, which increased the water surface temperature and influenced global weather patterns (heat dome globally and omega block in Europe) [15,16,17,18]. Furthermore, recent forecasts have shown that 2025 will become the third, or even the second, warmest year on record globally [15,16]. In 2024, central, eastern, and southeastern Europe experienced hot temperatures and low rainfall, resulting in extreme weather events like heatwaves and extreme drought in the southeast; these severe conditions greatly affected summer harvests in Hungary, Romania, Bulgaria, and Greece. Meanwhile, excessive rainfall in northern and northwestern Europe impacted the end of winter crops [19,20]. These agrometeorological conditions have led to yields falling below the five-year average for almost all crops in the European Union [19]. In Romania, the wheat harvest in 2024 had lower productivity but better quality compared to 2023. The extreme drought in June significantly impacted the wheat harvest, prompting the European Commission and the Romanian Government to provide compensatory payments and credit facilities to support the affected farmers [21,22].

This article investigates the microbiological and mycotoxicological contamination in common wheat in Romania and examines the impact of the weather conditions during the agricultural year 2023–2024. It also presents statistical correlations between contamination and the physical–chemical and colorimetric indicators in common wheat to see the impact on the quality of wheat crops and bakery products; the individual values of these indicators will be published in future articles. This is the first article to integrate some quality indicators of common wheat under extreme drought conditions, highlighting the importance of monitoring and mitigating these risks to ensure the safety and quality of wheat production. This information is valuable to growers, storekeepers, processors, grain traders, authorities and researchers in the agri-food field.

## 2. Results and Discussions

### 2.1. Agrometeorological Conditions in Romania in the Extremely Dry 2023–2024 Agricultural Year

**Figure 1:**
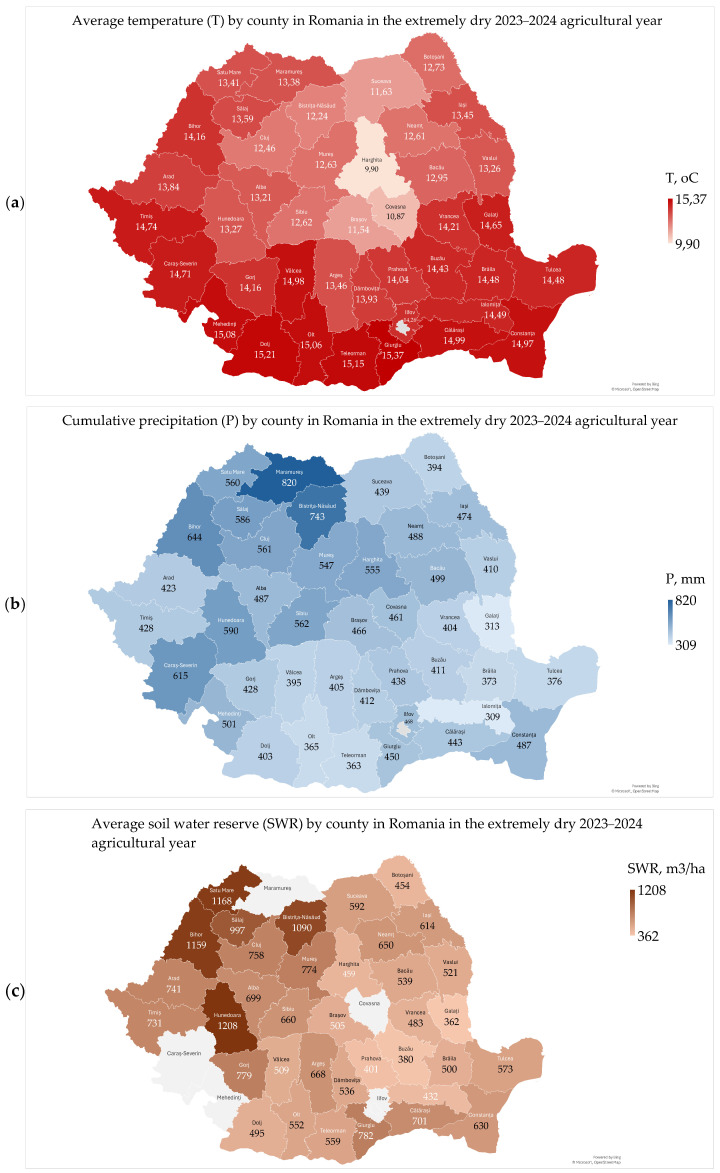
Agrometeorological conditions in Romania in the extremely dry 2023–2024 agricultural year: (**a**) average air temperature; (**b**) cumulative precipitation; (**c**) average soil water reserve.

The average air temperature ranged from 9.90 °C to 15.37 °C, with a mean of 13.67 °C and a median of 13.93 °C (Figure 1a) [23].

The cumulative precipitation ranged from 309 mm to 820 mm, with a mean of 476 mm and a median of 450 mm (Figure 1b) [23].

The average soil water reserve ranged from 362 m^3^/ha to 1208 m^3^/ha, with a mean of 657 m^3^/ha and a median of 603 m^3^/ha (Figure 1c) [23]. There are no data available for Maramures, Covasna, Caras-Severin, and Mehedinti counties.

The southern regions recorded the highest temperatures and the lowest cumulative precipitation and soil water reserve (Oltenia Plain, 15.12 °C, 423 mm, and 442 m^3^/ha; Southern Plain, 14.79 °C, 401 mm, and 365 m^3^/ha; Dobrogea, 14.73 °C, 431 mm, and 461 m^3^/ha; and the Southern Hilly Area, 14.24 °C, 443 mm, and 410 m^3^/ha), and the northern regions recorded lower temperatures and higher cumulative precipitation and soil water reserve (Transylvania, 12.34 °C, 580 mm, and 833 m^3^/ha; Moldavia, 13.19 °C, 428 mm, and 463 m^3^/ha; and the West Plain, 14.04 °C, 514 mm, and 959 m^3^/ha) (Figure 1a–c) [23]. May was wetter than average in Romania and Europe, and June, July and August were the warmest and driest, especially in southeastern Europe [15,16,17,18,23]. These extreme weather conditions have negatively affected the productivity and quality of wheat harvests, with farmers receiving governmental aid [21,22].

### 2.2. Microbiological and Mycotoxicological Contamination in Common Wheat in Romania in the Extremely Dry 2023–2024 Agricultural Year

#### 2.2.1. Moisture Content

**Figure 2:**
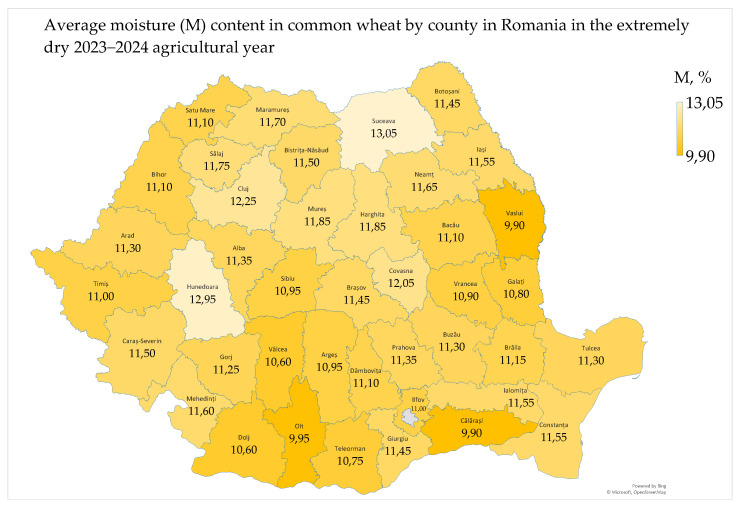
Average moisture content in common wheat in Romania in the extremely dry 2023–2024 agricultural year.

The moisture (M) content in common wheat ranged from 9.70% to 14.80%, with a mean of 11.30% and a median of 11.20%. The national wheat grading plan set a maximum permissible level of 14% moisture in wheat [24]. The average moisture content in common wheat by county ranged from 9.90% to 13.05% (Figure 2).

The moisture content in common wheat was appropriate in 98.8% (81/82) of the samples, with only one sample having an inappropriate value (>14%) [24]. Among these samples, 70 were very dry (<12%) and 11 were dry (12–14%). The inappropriate sample (semi-dry wheat) was collected from Hunedoara county, where the annual cumulative precipitation was 590 mm. The meteorological conditions, characterized by high temperatures and soil drought ranging from moderate to extreme, resulted in an average moisture content of 11.30% in common wheat in Romania. The southern and western regions (Oltenia Plain, Southern Plain, Southern Hilly Area, and West Plain) had below-average values due to their warmer climate and more alkaline soils, and the northern regions (Transylvania, and Moldavia) had above-average values due to their colder climate and more acidic soils [7,8]. The moisture content in common wheat in Dobrogea was favored by the rainy conditions during the harvest [23].

The moisture content in common wheat was significantly to very significantly correlated with air temperature, precipitation, and soil water reserve recorded in the extremely dry 2023–2024 agricultural year. Very significant correlations were observed in the West Plain and the Oltenia Plain, both regions having a sub-humid climate (Table S1) [23,25]. In Dobrogea, semi-dry wheat was correlated with very good hectoliter mass, a satisfactory Hagberg falling number (indicating high alpha-amylase content), very good protein content, satisfactory wet gluten content, and a good gluten index. Moisture content influenced the microbiological and mycotoxicological indicators (water activity, deoxynivalenol, and total aflatoxins) and decreased the red and yellow color of wheat (Table S1).

In Oltenia, very dry wheat was correlated with low hectoliter mass, high protein content, and high wet gluten content. Very low moisture content was very significantly correlated only with low water activity and negatively correlated with color indicators of common wheat (Table S1). In Transylvania and Moldavia, higher moisture content was correlated with higher water activity (Table S1), but these parameters were low in the extremely dry 2023–2024 agricultural year. The correlations highlight the effects of precipitation during the harvest period or the impact of extreme temperatures and drought on the physico-chemical, microbiological, mycotoxicological, and colorimetric quality indicators of common wheat [26,27,28].

The high temperatures and drought from June to August 2024 led to the harvest of common wheat with low moisture content. This resulted in the classification of grain as very dry wheat in the southeastern, southern, and western regions and as semi-dry wheat in the intra-Carpathian and northern regions. Precipitation at harvest affects the microbiological, mycotoxicological, physico-chemical, and colorimetric quality of common wheat, potentially causing post-harvest issues. Additionally, very dry wheat can cause difficulties during storage (the breaking of grains, a decrease in weight and quality, infection by insects, and a decrease in germination capacity), milling (increased energy consumption, a low flour extraction rate, a low quality of flour, and the increased wear and tear of grinding equipment), and baking. As a result, there are significant economic losses for farmers, storekeepers, millers and bakers, as well as a diminishing quality of finished foods.

#### 2.2.2. Water Activity

**Figure 3:**
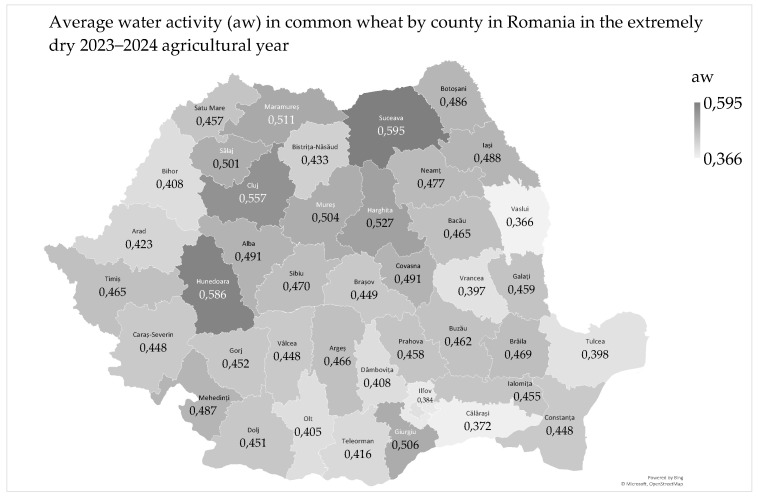
Average water activity in common wheat in Romania in the extremely dry 2023–2024 agricultural year.

The water activity (aw) in common wheat ranged from 0.318 to 0.722, with a mean of 0.463 and a median of 0.459. Most of the common wheat samples had water activity values ranging from 0.318 to 0.582 (96.3% of samples). The average water activity in common wheat by county ranged from 0.386 to 0.595 (Figure 3).

The average water activity in common wheat in Romania was 0.463, indicating a relatively low moisture level available for microbial and enzymatic activity. Water activity measures the water available for microorganisms and chemical processes, not just the total water content. At a water activity of 0.463, the risk of mold and bacteria development is reduced, which is beneficial for the long-term storage of wheat [29]. Even the highest detected value of water activity in common wheat (aw 0.722, in the Plopeni locality in Suceava county) was lower than the minimum value required for mycotoxin production. However, this level must be monitored to ensure the quality and stability of the wheat during storage and processing. Water activity is crucial in initiating fungal and mycotoxin contamination in grains from the field to storage. *Fusarium* spp. can develop in the field at aw 1.00, while *Aspergillus* spp. and *Penicillium* spp. develop post-harvest at aw < 0.95 [30]. Mycotoxin production is optimal at aw 0.996 and minimal at aw 0.80–0.82; intermediate values of aw 0.92–0.97 are critical post-harves as they can naturally initiate deoxynivalenol production [30,31,32,33].

The analysis of the geographical distribution of the average water activity in common wheat showed that the highest values were recorded in Transylvania (46–48° N, 23–26° E) and lower values were recorded in Moldavia, Southern Hilly Area, Oltenia Plain, West Plain, Southern Plain, and Dobrogea, which have higher aridity (Figure 3). Average water activity by soil had lower values in common wheat grown on neutral–alkaline soils (chernozem, aw 0.452) and higher values on acid soils (luvisol, aw 0.470; and phaeozem, aw 0.504) [34].

Water activity in common wheat significantly decreased in the Oltenia Plain and southern Moldavia due to high temperatures, rainfall deficit, and extreme drought from June to August 2024 and during the 2023–2024 agricultural year. In the West Plain, water activity was initially favored by June rainfall but decreased due to the rainfall deficit in July and August (Table S2) [23,35,36]. In Dobrogea, water activity was not correlated with the high temperatures and precipitation recorded during the harvest period, likely due to the dry conditions during the wheat ripening period (Table S2) [23]. This is supported by the fact that water activity was not correlated with total fungi, *Fusarium*-damaged kernels, and total aflatoxin mycotoxins in any region, but only with the deoxynivalenol mycotoxin in Dobrogea because of the delayed harvest (Table S2) [7,37].

Water activity influenced the physico-chemical properties of common wheat (Table S2). It was negatively correlated with moisture content, although this correlation is not always linear [38]. In the West Plain, water activity was highly significantly and positively correlated with protein content, wet gluten content, and the gluten index. Conversely, in the Oltenia Plain, which experienced extremely hot and dry conditions in May–August 2024, these correlations were highly significantly negative [23]. In the Southern Plain and Dobrogea, the extremely hot and dry conditions resulted in very low water activity and a very high Hagberg falling number of common wheat in 2024 [39]. Water activity values influence the chemical composition of wheat grain, as well as the fluidity, storage, and shelf life of wheat flour [29,40].

Water activity was significantly correlated with the color parameters (L*—whiteness; a*—redness; and b*—yellowness), leading to slight discoloration of common wheat in the West Plain, Southern Hilly Area, Dobrogea, and Oltenia Plain regions during the plant stress under drought conditions (Table S2) [36]. Increased moisture content and water activity in wheat decrease the development of color pigments [41,42].

The 2024 common wheat harvest shows favorable water activity values for long-term storage, reducing bacterial growth and mycotoxin production risks. However, the highest value requires monitoring to maintain wheat quality. Lower values were recorded in extra-Carpathian regions despite harvest precipitation, while higher values were observed in the intra-Carpathian region due to the cooler and wetter climate. High temperatures and extreme drought in June–August 2024 impacted water activity in the Oltenia Plain and southern Moldavia, and August precipitation delayed harvest in Dobrogea, affecting the microbiological, mycotoxicological, physicochemical, and colorimetric quality of the common wheat.

#### 2.2.3. Total Fungi

**Figure 4:**
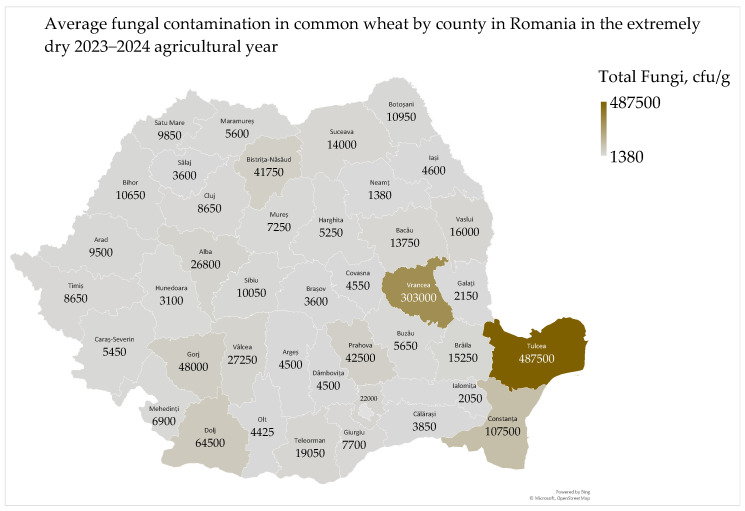
Average fungal contamination in common wheat in Romania in the extremely dry 2023–2024 agricultural year.

Total fungal contamination in common wheat ranged from 360 cfu to 960,000 cfu/g, with a mean of 34,466 cfu/g and a median of 6500 cfu/g. The average fungal contamination in common wheat by county ranged from 1380 cfu/g to 487,500 cfu/g (Figure 4).

The average fungal contamination in common wheat at harvest was 34,466 cfu/g, which is considered high and may pose risks to nutritional value and economic losses [43]. High temperatures and humidity favor fungal growth, while dry conditions can increase the frequency of functional genes in fungi like *Aspergillus* spp. [44]. Although there is no maximum permitted level for molds in common wheat at harvest, there are limits for molds in bread (100 cfu/g) and potentially toxic molds in animal feed (5000 cfu/g) [45,46]. Average fungal contamination was lower in the West Plain and Transylvania (47–48° N, 21–26° E) and higher in the Oltenia Plain (44° N, 24° E), Moldavia (47° N, 27° E), and Dobrogea (45° N, 28° E) (Figure 4). This distribution corresponds to lower fungal contamination in common wheat grown on acid soils (phaeozem, 7250 cfu/g; and luvisol, 13,889 cfu/g), and higher contamination on neutral–alkaline soils (chernozem, 64,797 cfu/g) [7,8,47,48].

Fungal contamination was influenced by agrometeorological conditions (Table S3). In Dobrogea, low soil water reserves favored fungal contamination, but high temperatures and precipitation did not (Table S3). Fungal growth depends on the type of fungi and agricultural practices [23,49]. In the Oltenia Plain, air temperature and precipitation in May significantly favored fungal contamination, while the lack of precipitation in July decreased it (Table S3) [23,36].

The influence of weather conditions in May on fungal contamination is supported by a positive and distinctly significant correlation with *Fusarium*-damaged kernels and a positive and highly significant correlation with the mycotoxin deoxynivalenol (Table S3) [7,8,23]. There were no significant correlations between fungal contamination and water activity or total aflatoxin contamination, indicating the inhibitory effect of the extremely dry weather in the summer of 2024 (Table S3) [9]. Although precipitation in May and early June favored contamination in common wheat, fungal growth was inhibited by the decrease in moisture content and water activity in wheat kernels caused by the extreme temperatures from June to August. In 2024, 96.3% of common wheat samples had water activity below the minimum value of 0.610, which is required for fungal growth [31].

Fungal contamination was negatively and significantly correlated with hectoliter mass, positively and significantly correlated with the Hagberg falling number in the Oltenia Plain, and positively correlated with wet gluten and the wet gluten deformation index in Dobrogea (Table S3). These correlations are a result of the meteorological conditions [39,50].

There were no significant correlations between fungal contamination and the color parameters (L*—whiteness; a*—redness; and b*—yellowness) of common wheat, indicating the inhibitory effect of the extremely dry weather in the summer of 2024 (Table S3) [36,49].

The total fungal contamination was high in the south of Moldavia and Dobrogea due to abundant precipitation during the harvest, which could lead to nutritional and economic problems in the post-harvest stages. The Oltenia Plain also had higher fungal contamination compared to the West Plain and Transylvania, where *Fusarium* fungi predominate [7,8]. Additionally, the correlation between fungal contamination and the incidence of *Fusarium*-damaged kernels and mycotoxin deoxynivalenol proves the complexity of wheat quality management. These findings emphasize the need for strategies to mitigate fungal contamination, especially in regions favorable for toxigenic fungi.

#### 2.2.4. Fusarium-Damaged Kernels

**Figure 5:**
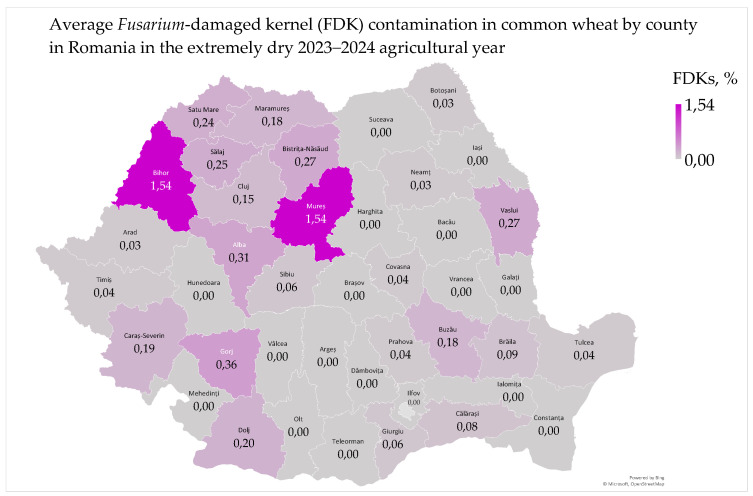
Average *Fusarium*-damaged kernel contamination in common wheat in Romania in the extremely dry 2023–2024 agricultural year.

*Fusarium*-damaged kernel (FDK) contamination in common wheat ranged from 0% to 2.96%, with a mean of 0.15% and a median of 0%. The national wheat grading plan includes three levels: grade I—maximum 0.3% (very good); grade II—maximum 0.5% (good); and grade III—maximum 1% (acceptable) and unacceptable—>1% (unacceptable for consumption) [8,24]. *Fusarium*-damaged kernel contamination had values above 1% only in two samples from West Plain (2.96%, in the Sacuieni locality in Bihor county) and Transylvania (2.92%, in the Sanpetru de Mures locality in Mures county). The average *Fusarium*-damaged kernel contamination in common wheat by county ranged from 0% to 1.54% (Figure 5).

The average incidence of *Fusarium*-damaged kernels in common wheat was 0.15%, which is considered low contamination, classifying the wheat as very good. *Fusarium* fungi growth is favored by moderate temperatures and high humidity, especially during the wheat flowering period in May and early June in Romania [7,8]. *Fusarium*-damaged kernel contamination was lower in Dobrogea, the Southern Plain, Moldavia, and the Oltenia Plain and higher in the West Plain, Southern Hilly Area, and Transylvania (Figure 5) [7,8]. This geographical distribution of *Fusarium*-damaged kernels in 2023–2024 corresponds to lower contamination in wheat grown on neutral–alkaline soils (chernozem, 0.07%) and higher contamination on acidic soils (luvisol, 0.15%; and phaeozem, 1.54%) [7,8,47,48].

*Fusarium*-damaged kernel contamination was influenced by agrometeorological conditions (Table S4). In May–June 2024, the Oltenia Plain, Southern Hilly Area, and West Plain experienced higher contamination due to warm and humid conditions (Table S4). They are the first regions of Romania to receive the influences of the Mediterranean climate in March–June [8,23,51]. In Dobrogea, precipitation in July and August increased contamination, as excess precipitation promotes *Fusarium* head blight disease, deoxynivalenol contamination and wheat sprouting [23,37,49,52].

*Fusarium*-damaged kernel contamination was not correlated with water activity in common wheat at harvest. However, it was correlated with total fungal contamination in the Oltenia Plain and deoxynivalenol contamination in the Southern Plain, the Oltenia Plain, the West Plain, Transylvania, and Moldavia regions that recorded precipitation in May (Table S4) [23]. Precipitation in May increased the water activity in the wheat grain and favored *Fusarium*-damaged kernel contamination, while the temperatures and extreme drought in June–August decreased the moisture content and water activity below the critical values for contamination (moisture > 14%, aw 0.92–0.97) [30,31,32,33]. In the West Plain, there were significant correlations between *Fusarium*-damaged kernels and total aflatoxin contamination, as *Aspergillus* spp. develop and produce aflatoxins in humid climates, even during dry years (Table S4) [5,8,9].

*Fusarium*-damaged kernel contamination negatively impacted the hectoliter mass and protein content of common wheat in both the West Plain and the Oltenia Plain (Table S4). In Moldavia, increased *Fusarium*-damaged kernel contamination led to a decrease in protein content and an increase in wet gluten content, adversely affecting the quality of wheat (Table S4) [53,54]. There were no significant correlations between *Fusarium*-damaged kernels and the wet gluten deformation index or the moisture content of common wheat at harvest, following the dry conditions of the summer of 2024 (Table S4) [23,55,56].

*Fusarium*-damaged kernel contamination showed significant correlations with color parameters (L*—whiteness; a*—redness; and b*—yellowness) in common wheat in Moldavia, the Southern Plain, and the West Plain, likely due to the wet conditions in the spring and the dry conditions in the summer of 2024 (Table S4) [23].

*Fusarium*-damaged kernel contamination in common wheat was favored by the precipitation in May–June. The extreme temperature and drought in June–August decreased the moisture content and water activity in wheat below the critical values for contamination. *Fusarium*-damaged kernel contamination decreased the physico-chemical, nutritional and colorimetric quality of the common wheat, which can affect the quality and price of the bread. Therefore, monitoring *Fusarium*-damaged kernel incidence is crucial to maintain wheat quality and safety.

#### 2.2.5. Deoxynivalenol Mycotoxin

**Figure 6:**
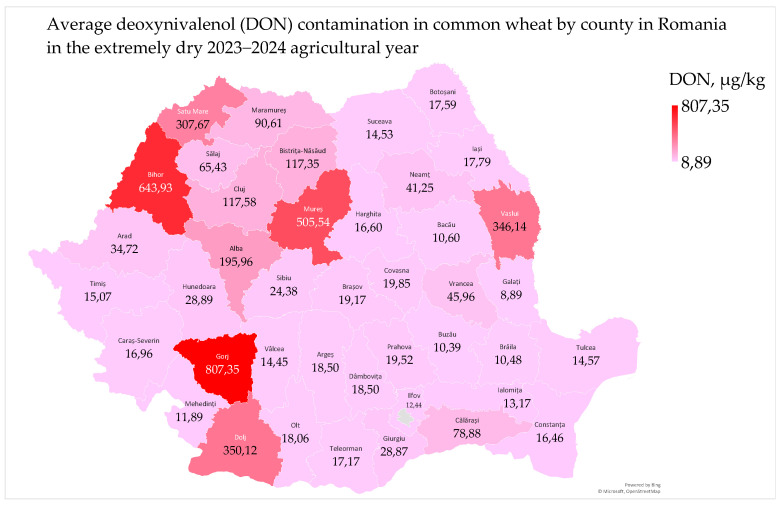
Average deoxynivalenol contamination in common wheat in Romania in the extremely dry 2023–2024 agricultural year.

Deoxynivalenol (DON) contamination in common wheat ranged from 8.62 µg/kg to 963.64 µg/kg, with a mean of 102.05 µg/kg and a median of <18.50 µg/kg. In 2024, the European Commission lowered the maximum permissible level of deoxynivalenol from 1250 µg/kg to 1000 µg/kg in unprocessed wheat and set a maximum level of 600 µg/kg for wheat milling products [57]. The average deoxynivalenol contamination in common wheat by county ranged from 8.89 µg/kg to 807.35 µg/kg (Figure 6).

The average deoxynivalenol contamination in common wheat in Romania was 102.05 µg/kg, which is considered low and safe for consumption according to international standards and previous years’ data [6,7,10]. The mycotoxin deoxynivalenol, produced by *Fusarium* fungi, develops in moderate temperatures and high humidity during May–June, the critical wheat flowering period in Romania [6,7,8]. The highest deoxynivalenol levels were found in counties with the most *Fusarium*-damaged kernels in common wheat (Figure 5 and Figure 6) [7,8]. However, the presence of deoxynivalenol in wheat samples with 0% FDKs highlights the need for instrumental methods (near-infrared spectroscopy, digital imaging seed phenotyping, etc.) in detecting *Fusarium* fungi, especially in dry conditions [58].

Deoxynivalenol contamination in common wheat was lower in Dobrogea, the Southern Plain, Moldavia, and the Oltenia Plain and higher in Transylvania, the Southern Hilly Area, and the West Plain. The highest contamination was found in the northwest of the country, between 47–48° N and 22–25° E (Figure 6) [6,7,8,10,23,25]. Additionally, deoxynivalenol contamination was lower in wheat grown on neutral–alkaline soils (chernozem, 67.33 µg/kg) and higher in wheat grown on acidic soils (luvisol, 110.17 µg/kg; and phaeozem, 505.54 µg/kg) [7,8]. The distribution of deoxynivalenol and *Fusarium*-damaged kernels in common wheat in 2023–2024 is similar to the distribution of deoxynivalenol in 2012–2014 and *Fusarium*-damaged kernels in 2015–2016 [7,8].

Deoxynivalenol contamination in common wheat was significantly influenced by agrometeorological parameters (Table S5). In the Oltenia Plain, moderate temperatures and precipitation in May favored deoxynivalenol contamination, while deficient precipitation in July and high temperatures in August inhibited it. In the Southern Hilly Area, deoxynivalenol contamination was not influenced by air temperature but was favored by May precipitation and soil water reserve in May and June and decreased by the June–July precipitation deficit. In the Southern Plain, high temperature and deficient precipitation in May and the soil water deficit in March and June inhibited deoxynivalenol contamination. In the West Plain, moderate temperatures in June, June and annual precipitation, and March–June and annual soil water reserves favored deoxynivalenol contamination. The analysis of geographic distributions of statistical correlations showed that deoxynivalenol contamination in common wheat grown in the Oltenia Plain, the Southern Hilly Area, and the Southern Plain was influenced by Mediterranean air masses in May–June, and in the West Plain, it was influenced by the abundant precipitation in June [7,8,23].

Deoxynivalenol contamination was correlated with microbiological and mycotoxicological parameters in common wheat (Table S5). In Dobrogea, deoxynivalenol contamination and water activity were very low but significantly correlated due to the precipitation during harvest (Table S5) [7,37]. The production of deoxynivalenol is minimal at water activity levels of 0.90–0.91 and optimal at 0.98–0.99 [30,31,32,33]. Deoxynivalenol contamination and *Fusarium*-damaged kernels were distinctly and significantly correlated in the Southern Hilly Area and highly and significantly correlated in the Southern Plain, the Oltenia Plain, the West Plain, Transylvania, and Moldavia. The correlations of deoxynivalenol with *Fusarium*-damaged kernels and agroclimatic parameters have the same geographical distribution (Figure 5 and Figure 6; Tables S4 and S5) [7,8,23]. The correlation of deoxynivalenol and total aflatoxin mycotoxins in the West Plain and the correlation of deoxynivalenol and total fungi in the Oltenia Plain were favored by soil water reserve in May–June and decreased by high temperatures in June–August (Tables S5 and S6) [9,10,23].

Deoxynivalenol contamination was negatively correlated with physico-chemical parameters in common wheat (Table S5). It decreased hectoliter mass in the West Plain and Oltenia Plain and reduced the wet gluten content and gluten index in the West Plain. Additionally, deoxynivalenol contamination lowered protein and wet gluten contents in Moldavia (Table S5) [39,53,59].

Deoxynivalenol contamination was correlated with sensory–colorimetric parameters of common wheat (Table S5). In the West Plain, it was correlated with color parameters (L*—whiteness; a*—redness; and b*—yellowness), indicating that wheat color was affected under wet spring and dry summer conditions in 2024 (Table S5) [39,53].

The deoxynivalenol contamination levels varied significantly across different regions, with lower values in the extra-Carpathian regions and higher values in the intra-Carpathian region. Moderate temperatures and precipitation in May–June favored contamination in the northern regions, while high temperatures and deficient precipitation inhibited it in the southern regions. The study also observed correlations between deoxynivalenol contamination and various microbiological, mycotoxicological, physico-chemical, and colorimetric parameters in common wheat. These findings highlight the importance of monitoring and managing agrometeorological conditions to control deoxynivalenol contamination, ensuring wheat quality and safety during storage, milling, and baking processes. This is particularly crucial for the agro-food sector in the context of climate change.

The current and projected climate changes until 2100 indicate an increase in the occurrence and severity of contamination with *Fusarium* fungi and the mycotoxin deoxynivalenol in wheat across the northern, northwestern, and eastern regions of Europe, extending to Western Siberia in Russia [8,60,61].

#### 2.2.6. Total Aflatoxin Mycotoxins

**Figure 7:**
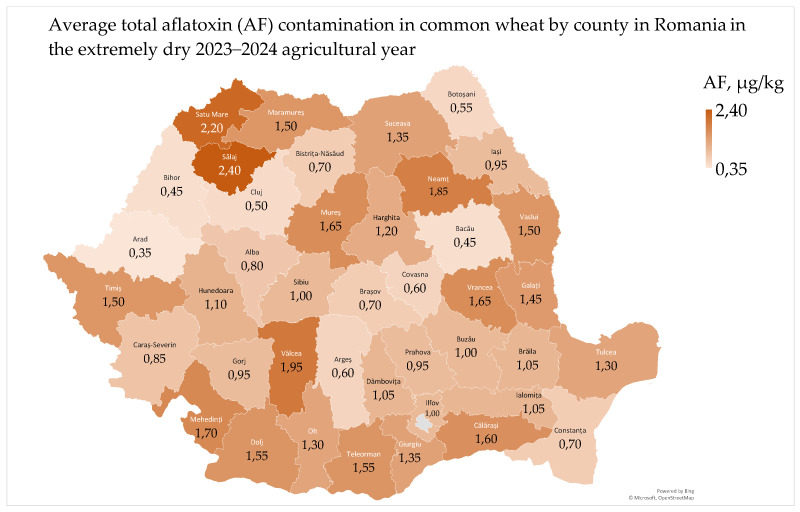
Total aflatoxin contamination in common wheat in Romania in the extremely dry 2023–2024 agricultural year.

Total aflatoxin (AF) contamination in common wheat ranged from 0 µg/kg to 3.62 µg/kg, with a mean of 1.12 µg/kg and a median of 1.05 µg/kg. The European Commission has set a maximum permissible limit of 4.0 μg/kg for total aflatoxins (the sum of B1, B2, G1 and G2) and 2.0 μg/kg for aflatoxin B1 in all cereals and cereal products, including processed cereal products [62]. The average total aflatoxin contamination in common wheat by county ranged from 0.35 µg/kg to 2.40 µg/kg (Figure 7).

The average aflatoxin contamination of 1.12 µg/kg in common wheat at harvest is considered very low, but it may pose a health risk with long-term exposure [6,9]. Aflatoxins are highly hepatogenic, neurotoxic, immunosuppressive and carcinogenic (Group 1) [63,64]. In 2024, the average total aflatoxin contamination in common wheat in Romania was lower in Dobrogea, Transylvania, the West Plain, and the Southern Hilly Area but higher in the Southern Plain, Moldavia, and Oltenia Plain regions. The highest average values were recorded at 44° N and 47–48° N and 23–24° E, 29° E, where high temperatures and precipitation occurred in June and July. Warm and humid climates favor aflatoxin contamination, with *A. flavus* and *A. parasiticus* growing at temperatures of 20–35 °C and water activity > 0.90 [65,66].

The highest individual values of aflatoxin in common wheat were recorded in the northwest (3.62 µg/kg, in Salaj county; 2.04 µg/kg, in Maramures county; and 2.48 µg/kg, in Satu Mare county) and in the west (2.17 µg/kg, in Timis county) of Romania. These areas are located in the Pannonian Basin and have a climate similar to Central Europe (Figure 7). Due to climate change, contamination with *Aspergillus* spp. and aflatoxins has become common in this temperate European region; however, contamination is more common in tropical and subtropical areas (25–35° N/S) [5,9,66]. Additionally, aflatoxin contamination had lower values in wheat grown on luvisol (1.10 µg/kg) and chernozem (1.12 µg/kg) and higher values on phaeozem (1.63 µg/kg). This demonstrates the ability of *Aspergillus* spp. to grow on all types of agricultural soils [9,67,68].

Aflatoxin contamination in common wheat showed few correlations with agrometeorological parameters (Table S6). It was not correlated with air temperature in any agricultural region. In the West Plain, aflatoxin contamination was favored by precipitation deficit in May and annually and soil water reserve in June (Table S6) [69].

Aflatoxin contamination was correlated with microbiological and mycotoxicological indicators in common wheat (Table S6). Specifically, it was correlated with *Fusarium*-damaged kernels and deoxynivalenol in the West Plain due to precipitation deficit and higher soil water reserve (Table S6) [9,69,70]. There was no correlation between total aflatoxin contamination and water activity or total fungi in any agricultural region (Table S6). The maximum water activity (aw 0.722) in common wheat in the extremely dry 2023–2024 year was below the minimum 0.78–0.84 required for aflatoxin production [30].

Total aflatoxin contamination showed few correlations with physico-chemical indicators in common wheat (Table S6). Specifically, aflatoxin contamination was correlated with moisture content in the West Plain and had negative effects on the hectoliter mass in Transylvania. However, there was no correlation between aflatoxin contamination and the Hagberg falling number (alpha-amylase content), protein, wet gluten, the wet gluten deformation index, and the gluten index in common wheat. It is important to note that the physico-chemical indicators in common wheat in Transylvania and the West Plain are influenced by both weather conditions and environmental pollution with heavy metals [9,10].

Total aflatoxin contamination showed no significant correlations with color parameters (L*—whiteness; a*—redness; and b*—yellowness) in common wheat (Table S6). Contamination with *Aspergillus* fungi can influence wheat color, and some colorimetric methods for early detection have been developed [71].

The average of total aflatoxin contamination in common wheat varied across regions, with higher levels in extra-Carpathian regions and lower levels in humid climates, particularly in northwest and west Romania. While few correlations were found between aflatoxin contamination and various agrometeorological, microbiological, and physico-chemical indicators, aflatoxin contamination was associated with *Fusarium*-damaged kernels and the mycotoxin deoxynivalenol in the West Plain. Aflatoxin contamination in wheat is more likely in warm and humid storage conditions and can be distributed throughout flour during milling. Although baking can reduce aflatoxin levels, it may not eliminate them, making early-stage control crucial for food safety.

Although total aflatoxin contamination in common wheat was low, maize experienced very high contamination during the extremely dry 2023–2024 agricultural year. Romania, Bulgaria, Serbia, and Hungary reported an aflatoxin B1 contamination of up to 506 ppb in maize, leading to refused exports [72,73].

Current and projected climate changes until 2100 indicate an increase in the *Aspergillus* spp. and aflatoxin contamination in wheat and maize from the southern to the northern regions of Europe due to increased drought [5,9,74,75]. The increase in fungal and mycotoxin contamination in cereals during extreme weather events (heavy rainfall, floods, heatwaves, and drought) calls for measures to combat the effects of climate change. These extreme events are becoming more common in Europe and have significant environmental, social, and economic consequences [5,6,7,8,9,10,74,76].

## 3. Conclusions

The weather conditions in 2023–2024, influenced by climate change and global atmospheric systems, significantly impacted the production and quality of cereal crops in Europe.

This study highlights the impact of agrometeorological conditions on the microbiological and mycotoxicological quality of common wheat in Romania during the extremely dry 2023–2024 agricultural year. Wheat from regions with moderate temperatures and precipitation showed higher contamination levels, while wheat from areas with extreme temperatures and drought had lower contamination. Despite low overall contamination, the quality of common wheat and finished foods can be negatively affected, leading to potential economic losses. The coexistence of deoxynivalenol and total aflatoxins in wheat grown in the northwest of the country indicates the spread of contamination due to dry conditions and climate change. The results are crucial in the context of extreme weather events and climate change, as they lead to increased contamination with fungi and mycotoxins in common wheat in Europe.

This research study will continue in two publications focusing on the physico-chemical and sensory–colorimetric quality of common wheat in Romania during the extremely dry 2023–2024 agricultural year. These publications will have a synergistic approach with the present article.

## 4. Materials and Methods

### 4.1. Agrometeorological Data

The agrometeorological parameters (air temperature, °C; precipitation, mm; soil water reserve, m^3^/ha) were recorded in the period 1 September 2023–31 August 2024, by the official network of agrometeorological stations of the National Meteorological Administration, Meteo-Romania [23].

The annual values of the agrometeorological parameters in Romania in the extremely dry 2023–2024 agricultural year, are presented in Figure 1a–c.

### 4.2. Sampling of Common Wheat

Common wheat was sampled (*N* = 82; 2 samples/county; 3 kg/sample) by the County Agricultural Directorates of the Ministry of Agriculture and Rural Development (MARD). The sampling procedure is detailed in [7,8].

The counties and agricultural regions in Romania are delimited in Figure 1, Figure 2, Figure 3, Figure 4, Figure 5, Figure 6, Figure 7 and Figure 8 and the agricultural regions are named in Figure 8.

### 4.3. Analysis of Common Wheat

Common wheat samples were analyzed using methods accredited by the Romanian Accreditation Association (RENAR) according to the standard SR EN ISO/IEC 17025:2018 [77].

The moisture content (M, %) was determined following the standard SR EN ISO 712:2010 [78] and using a laboratory oven MRC DK-500 WT (MRC Ltd., Holon, Israel).

Water activity (aw) was determined following the procedure of Aquaspector AQS 31 and using an Aquaspector AQS 31 (Nagy Messsysteme GmbH, Gäufelden, Germany).

Total fungi (cfu/g) were determined following the standard SR ISO 21527-2/2009 [79] and using a thermostat Panasonic MIR-154-PE with cooling and forced ventilation at 25 °C (PHC Europe B.V., Breda, The Netherlands).

*Fusarium*-damaged kernels (FDKs, %) were determined following the standard SR EN ISO 7970:2011 [80] by a visual method.

Deoxynivalenol (DON, µg/kg) was determined following the procedure of Ridascreen^®^ DON (R-Biopharm, Darmstadt, Germany) and using a Sunrise™ plate reader at 450 nm (Tecan Group Ltd., Männedorf, Switzerland).

Total aflatoxins (AF, µg/kg) were determined following the procedure of Ridascreen^®^ Aflatoxin Total (R-Biopharm, Darmstadt, Germany) and using a Sunrise™ plate reader at 450 nm (Tecan Group Ltd., Männedorf, Switzerland).

The average values of the microbiological and mycotoxicological parameters by county and agricultural region are presented in Figure 2, Figure 3, Figure 4, Figure 5, Figure 6 and Figure 7.

### 4.4. Statistical Analysis

Analytical data were collected in a database and statistically evaluated with JASP Team version 0.17.1 software (University of Amsterdam, Amsterdam, The Netherlands). The linear correlations between the microbiological, mycotoxicological, physico-chemical, sensory–colorimetric and agrometeorological parameters were calculated for each agricultural region; three thresholds were used to interpret the significance: significant correlation * (*p*-value < 0.05), distinctly significant correlation ** (*p*-value < 0.01) and highly significant correlation *** (*p*-value < 0.001). The datasets used to calculate the correlations were relatively equal in size and extrapolations were used when there were differences. The accuracy of these extrapolations was checked using the bootstrapping method applied to estimate the confidence interval for the correlations.

Pearson correlation coefficients are presented in Tables S1–S6 in the Supplementary Materials.

This article presents Pearson correlations between microbiological and mycotoxicological parameters with the physico-chemical and sensory–colorimetric parameters of common wheat:

(a) Physico-chemical parameters: hectoliter mass (HM, kg/hectolitre), Hagberg falling number (HFN, seconds), protein (P, % dry matter), wet gluten (WG, %), wet gluten deformation index (WGDI, mm), gluten index (GI). Specific equipment was utilized for each determination. The laboratory methods are accredited by the RENAR.
(b) Sensory–colorimetric parameters: L*—sample brightness on a scale from 0 to 100 (L* = 0, black; L* = 100, white); a*—sample color on a scale from pure green to pure red (−a, green; +a, red); and b*—sample color on a scale from pure blue to pure yellow (−b, blue; +b, yellow). Parameters were determined with a CM-5 spectrophotometer (Konica Minolta, Tokyo, Japan). The colorimetric indicators of Romanian wheat fall within the spectrum towards white, red and yellow.

### 4.5. Geographic Distribution

The geographic distributions of agrometeorological, microbiological and mycotoxicological parameters in 2023–2024 were assessed by Microsoft 365 Business Standard software (Microsoft, Redmond, Washington, DC, USA) (Figure 1, Figure 2, Figure 3, Figure 4, Figure 5, Figure 6 and Figure 7).

## Figures and Tables

**Figure 8:**
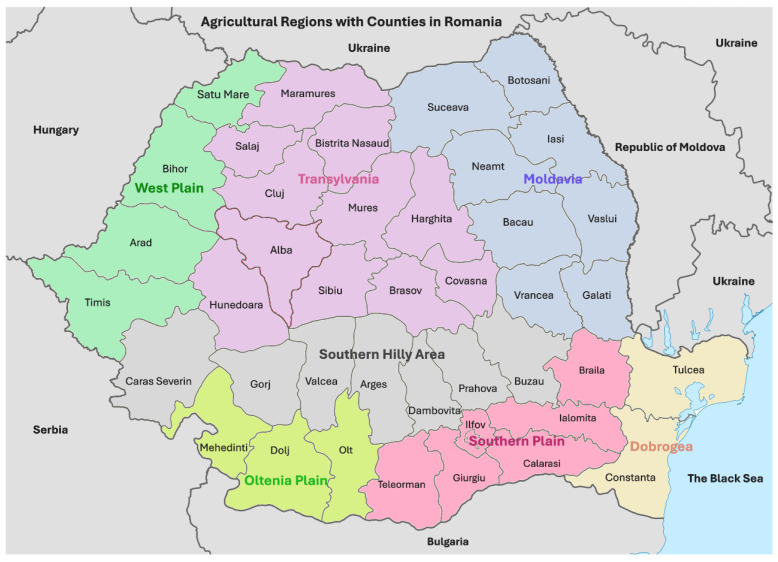
Agricultural regions with counties in Romania (45°56′39.43″ N and 25°00′33.95″ E).

## Data Availability

The original contributions presented in this study are included in the article and Supplementary Material. Further inquiries can be directed to the corresponding authors.

## Supplementary Materials

The following are available online at https://www.mdpi.com/article/10.3390/toxins17040154/s1. Table S1. Pearson correlation of moisture content with quality indicators in common wheat and the agrometeorological parameters by region in Romania in the extremely dry 2023–2024 agricultural year; Table S2. Pearson correlation of water activity with quality indicators in common wheat and the agrometeorological parameters by region in Romania in the extremely dry 2023–2024 agricultural year; Table S3. Pearson correlation of total fungal contamination with quality indicators in common wheat and the agrometeorological parameters by region in Romania in the extremely dry 2023–2024 agricultural year; Table S4. Pearson correlation of *Fusarium*-damaged kernels contamination with quality indicators in common wheat and the agrometeorological parameters by region in Romania in the extremely dry 2023–2024 agricultural year; Table S5. Pearson correlation of deoxynivalenol contamination with quality indicators in common wheat and the agrometeorological parameters by region in Romania in the extremely dry 2023–2024 agricultural year; Table S6. Pearson correlation of total aflatoxin contamination with quality indicators in common wheat and the agrometeorological conditions by region in Romania in the extremely dry 2023–2024 agricultural year.
