# Supplementary material for: Microbiological and Mycotoxicological Quality of Common Wheat in Romania in the Extremely Dry 2023–2024 Agricultural Year
Source: Toxins (Basel). 2025 Mar 22;17(4):154. doi: 10.3390/toxins17040154 (PMC12030849; doi:10.3390/toxins17040154)
Supplement: Supplementary file 1 [file toxins-17-00154-s001.zip › toxins-3491964-supplementary.pdf]

Supplementary Material: Microbiological and Mycotoxicological Quality of Common Wheat in Romania in the Extremely Dry 2023–2024 Agricultural Year

Table S1. Pearson correlation of moisture content with quality indicators in common wheat and the agrometeorological parameters by region in Romania in the extremely dry 2023–2024 agricultural year.

Pearson Correlation (r_{xy}) of Average Moisture (M) Content with the Average Quality Indicators in Common Wheat and the Average Agrometeorological Parameters by Agricultural Region in Romania in the Extremely Dry 2023–2024 Agricultural Year									
Variable			Transylvania	Southern Hilly Area	West Plain	Oltenia Plain	Moldavia	Southern Plain	Dobrogea
Quality Indicators in Common Wheat	Microbiological and Mycotoxicological	Water Activity, aw	0.843 ***	0.416 *	0.457 *	0.927 ***	0.971 ***	0.782 ***	0.543 **
		Total Fungi	-0.086	-0.071	-0.146	0.009	-0.218	-0.019	-0.075
		Fusarium-damaged kernel, FDK	-0.090	-0.023	-0.031	-0.080	-0.388	-0.253	N.a.
		Deoxynivalenol, DON	-0.086	0.115	-0.120	0.073	0.286	-0.596 **	-0.404
		Total Aflatoxin, AF	-0.159	0.056	-0.530 *	0.106	0.165	-0.212	-0.420
	Physico-chemical	Hectolitre Mass, HM	0.282	0.001	0.388	-0.436 *	0.240	0.133	0.262
		Hagberg Falling Number, HFN	0.083	-0.193	-0.040	-0.329	-0.351	-0.461 *	-0.968 ***
		Protein, P	-0.444 *	-0.110	0.079	-0.679 ***	0.040	-0.088	-0.856 ***
		Wet Gluten, WG	-0.384	-0.054	-0.003	-0.649 ***	-0.047	-0.175	-0.761 ***
		Wet Gluten Deformation Index, WGDI	-0.020	-0.359	-0.095	-0.194	-0.066	0.110	0.428 *
		Gluten Index, GI	0.187	0.377	-0.350	-0.397	0.146	0.358	0.788 ***
	Sensory–colorimetric	L*—whiteness	0.065	0.378	0.342	0.833 ***	0.028	0.019	-0.891 ***
		a*—redness	-0.052	-0.513 *	-0.148	-0.753 ***	-0.173	0.008	-0.762 ***
		b*—yellowness	-0.087	-0.496 *	-0.295	-0.880 ***	0.348	0.017	-0.916 ***
Agrometeorological Parameters	Air Temperature	May 2024	0.003	0.189	-0.679 ***	-0.177	-0.428 *	0.337	0.556 **
		June 2024	-0.032	-0.332	-0.276	-0.956 ***	-0.550 **	0.173	0.556 **
		July 2024	0.026	-0.178	-0.322	0.912 ***	-0.580 **	0.208	0.557 **
		August 2024	0.107	-0.063	-0.185	-0.372	-0.563 **	0.279	0.555 **
		Annual, September 2023–August 2024	0.014	0.059	-0.423 *	0.028	-0.550 **	0.267	-0.558 **
	Precipitation	May 2024	0.437 *	0.232	0.700 ***	0.743 ***	0.321	0.481 *	0.553 **
		June 2024	-0.162	0.291	0.235	0.934 ***	-0.048	0.430 *	0.562 **
		July 2024	-0.224	0.265	-0.715 ***	0.315	0.399	0.487 *	0.553 **
		August 2024	-0.282	0.272	0.631 **	-0.883 ***	-0.283	-0.249	0.558 **
		Annual, September 2023–August 2024	-0.018	0.407	-0.112	0.954 ***	0.216	0.580 *	0.556 **
	Soil Water Reserve	Sepember 2023 (0–20 cm)	0.607 ***	-0.230	-0.302	-0.861 ***	0.616 **	0.214	0.556 **
		October 2023 (0–20 cm)	0.307	-0.092	-0.265	-0.815 ***	0.650 **	-0.081	0.556 **
		November 2023 (0–50 cm)	0.362	-0.014	-0.485 *	-0.852 ***	-0.049	0.103	0.556 **
		March 2024 (0–100 cm)	0.329	-0.243	-0.205	-0.859 ***	0.090	0.377	0.556 **
		April 2024 (0–100 cm)	0.360	0.028	0.023	-0.809 ***	0.551 **	0.090	-0.556 **
		May 2024 (0–100 cm)	0.378	0.086	0.156	-0.713 ***	0.304	0.266	-0.556 **
		June 2024 (0–100 cm)	0.306	0.247	-0.128	-0.785 ***	-0.028	0.512 *	-0.556 **
		Annual, September 2023–June 2024	0.327	0.124	-0.287	-0.593 **	0.326	0.231	-0.228

Significance (two-tailed): * *p*-value < 0.05 – significant differences, ** *p*-value < 0.01 – distinct significant differences, *** *p*-value < 0.001 – very significant differences.

Table S2. Pearson correlation of water activity with quality indicators in common wheat and the agrometeorological parameters by region in Romania in the extremely dry 2023–2024 agricultural year.

Pearson Correlation (r_{xy}) of Water Activity (aw) with the Average Quality Indicators in Common Wheat and the Average Agrometeorological Parameters by Agricultural Region in Romania in the Extremely Dry 2023–2024 Agricultural Year									
**Variable**			**Transylvania**	**Southern Hilly Area**	**West Plain**	**Oltenia Plain**	**Moldavia**	**Southern Plain**	**Dobrogea**
**Quality Indicators in Common Wheat**	**Microbiological and Mycotoxicological**	**Moisture, M**	0.843 ***	0.416 *	-0.457 *	0.927 ***	0.971 ***	0.782 ***	0.543 **
		**Total Fungi**	-0.172	0.065	0.079	0.018	-0.257	-0.100	0.077
		***Fusarium*-damaged kernel, FDK**	-0.057	-0.206	-0.401	0.024	-0.392	0.076	N.a.
		**Deoxynivalenol, DON**	-0.038	0.041	-0.306	-0.059	-0.303	-0.341	-0.965 ***
		**Total Aflatoxin, AF**	-0.019	-0.228	0.064	0.029	0.065	-0.249	-0.320
	**Physico-chemical**	**Hectolitre Mass**	0.266	-0.119	0.399	-0.414	0.310	0.126	-0.644 **
		**Hagberg Falling Number, HFN**	-0.006	-0.065	-0.310	-0.324	-0.291	-0.512 *	-0.527 *
		**Protein, P**	-0.333	-0.014	0.509 *	-0.630 **	0.086	-0.305	-0.201
		**Wet Gluten, WG**	-0.264	-0.156	0.5770 **	-0.634 **	0.033	-0.409	-0.151
		**Wet Gluten Deformation Index, WGDI**	-0.022	0.103	-0.016	-0.034	-0.007	-0.174	0.230
		**Gluten Index, GI**	0.033	0.128	0.872 ***	-0.313	0.099	0.364	-0.078
	**Sensory–colorimetric**	**L*—whiteness**	-0.07	0.197	-0.613 **	0.699 ***	0.032	0.044	-0.740 ***
		**a*—redness**	-0.059	-0.484 *	0.429 *	-0.729 ***	-0.123	-0.028	-0.488 *
		**b*—yellowness**	-0.099	-0.424 *	0.456 *	-0.837 ***	-0.303	-0.010	-0.548 **
**Agrometeorological Parameters**	**Air Temperature**	**May 2024**	0.092	-0.117	0.417	-0.070	-0.372	0.088	-0.094
		**June 2024**	0.073	-0.092	0.221	-0.787 ***	-0.521 *	-0.347	-0.094
		**July 2024**	0.088	0.038	0.258	-0.725 ***	-0.557 **	-0.287	-0.094
		**August 2024**	0.148	0.231	0.029	-0.381	-0.547 **	-0.272	-0.092
		**Annual, September 2023–August 2024**	0.102	-0.067	0.138	0.097	-0.522 *	-0.276	-0.096
	**Precipitation**	**May 2024**	0.450 *	0.121	-0.514 *	0.659 **	0.233	-0.047	0.094
		**June 2024**	-0.297	-0.395	-0.586 **	0.749 ***	-0.212	0.711 ***	0.092
		**July 2024**	-0.072	-0.071	0.789 ***	0.184	0.388	0.578 *	0.102
		**August 2024**	-0.345	0.231	0.826 ***	0.708 ***	-0.251	-0.020	0.093
		**Annual, September 2023–August 2024**	-0.016	-0.067	-0.361	0.776 ***	0.120	0.645 **	0.094
	**Soil Water Reserve**	**Sepember 2023 (0–20 cm)**	0.520 *	0.033	0.329	0.937 ***	0.527 *	0.181	0.094
		**October 2023 (0–20 cm)**	0.180	0.237	0.315	0.902 ***	0.624 **	-0.121	0.094
		**November 2023 (0–50 cm)**	0.175	-0.115	0.336	0.931 ***	-0.016	-0.025	0.094
		**March 2024 (0–100 cm)**	0.102	-0.044	-0.164	0.935 ***	0.070	0.029	0.094
		**April 2024 (0–100 cm)**	0.171	0.142	-0.402	0.897 ***	0.498 *	-0.304	0.094
		**May 2024 (0–100 cm)**	0.205	0.198	-0.496 *	0.817 ***	0.187	-0.139	0.094
		**June 2024 (0–100 cm)**	0.057	-0.269	-0.215	0.881 ***	-0.151	0.025	0.094
		**Annual, September 2023–June 2024**	0.104	0.073	-0.122	0.600 ***	0.223	-0.130	0.094

Significance (two-tailed): * *p*-value < 0.05 – significant differences, ** *p*-value < 0.01 – distinct significant differences, *** *p*-value < 0.001 – very significant differences.

Table S3. Pearson correlation of total fungal contamination with quality indicators in common wheat and the agrometeorological parameters by region in Romania in the extremely dry 2023–2024 agricultural year.

Pearson Correlation (r_{xy}) of Average Total Fungal Contamination with the Average Quality Indicators in Common Wheat and the Average Agrometeorological Parameters by Agricultural Region in Romania in the Extremely Dry 2023–2024 Agricultural Year

Variable			Transylvania	Southern Hilly Area	West Plain	Oltenia Plain	Moldavia	Southern Plain	Dobrogea
Quality Indicators in Common Wheat	Microbiological and Mycotoxicological	Moisture, M	-0.086	-0.071	-0.146	0.009	-0.218	-0.019	-0.075
		Water Activity, aw	-0.172	0.065	0.079	0.018	-0.257	-0.100	0.077
		Fusarium-damaged kernel, FDK	0.052	0.034	0.076	0.669 ***	0.273	-0.097	NaN
		Deoxynivalenol, DON	0.077	0.201	-0.003	0.612 **	0.252	-0.175	-0.196
		Total Aflatoxin, AF	-0.163	0.114	0.058	0.035	0.090	-0.263	0.207
	Physico-chemical	Hectolitre Mass, HM	0.107	-0.417	-0.234	-0.582 **	-0.160	-0.119	-0.263
		Hagberg Falling Number, HFN	-0.086	-0.295	-0.020	0.620 **	0.095	0.106	-0.083
		Protein, P	-0.117	-0.095	-0.167	-0.418	-0.060	0.074	0.366
		Wet Gluten, WG	-0.155	-0.128	-0186	-0.361	-0.245	0.078	0.456 *
		Wet Gluten Deformation Index, WGDI	0.077	0.106	0.083	0.001	-0.155	0.212	0.532 *
		Gluten Index, GI	0.129	0.079	0.027	0.336	0.085	-0.163	-0.092
	Sensory–colorimetric	L*—whiteness	0.121	0.147	0.045	0.122	0.023	0.216	-0.181
		a*—redness	-0.174	-0.166	-0.133	-0.213	-0.065	-0.251	-0.356
		b*—yellowness	-0.019	-0.174	-0.027	0.107	0.108	-0.224	-0.185
Agrometeorological Parameters	Air Temperature	May 2024	0.116	0.091	-0.013	0.665 **	0.179	0.084	-0.533 *
		June 2024	0.124	0.333	-0.007	-0.075	0.263	0.033	-0.534 *
		July 2024	0.087	0.397	-0.006	0.168	0.261	0.020	-0.533 *
		August 2024	0.070	0.330	-0.028	-0.639 **	0.271	0.042	-0.534 *
		Annual, September 2023–August 2024	0.077	0.102	-0.035	0.690 ***	0.254	0.048	-0.533 *
	Precipitation	May 2024	0.084	0.086	-0.021	0.467 *	-0.047	0.127	-0.536 *
		June 2024	0.318	-0.063	-0.049	-0.102	0.108	-0.070	-0.530 *
		July 2024	-0.032	-0.178	0.039	-0.632 **	-0.114	-0.005	-0.528 *
		August 2024	0.202	0.295	-0.053	0.255	0.153	0.133	-0.533 *
		Annual, September 2023–August 2024	0.196	-0.131	0.052	-0.007	0.014	0.027	-0.536 *
	Soil Water Reserve	Sepember 2023 (0–20 cm)	0.231	0.027	0.034	0.202	-0.099	-0.327	-0.533 *
		October 2023 (0–20 cm)	0.165	0.303	0.035	0.263	-0.267	0.046	-0.533 *
		November 2023 (0–50 cm)	0.090	0.039	0.017	0.216	-0.290	0.203	-0.533 *
		March 2024 (0–100 cm)	0.151	-0.180	-0.029	0.207	0.029	0.220	-0.533 *
		April 2024 (0–100 cm)	0.136	-0.101	-0.044	0.270	-0.051	0.249	-0.533 *
		May 2024 (0–100 cm)	0.201	-0.118	-0.046	0.363	-0.018	0.136	-0.533 *
		June 2024 (0–100 cm)	0.323	-0.077	-0.029	0.291	-0.002	0.124	-0.533 *
		Annual, September 2023–June 2024	0.224	-0.092	0.010	0.192	-0.126	0.260	-0.533 *

Significance (two-tailed): * *p*-value < 0.05 – significant differences, ** *p*-value < 0.01 – distinct significant differences, *** *p*-value < 0.001 – very significant differences.

Table S4. Pearson correlation of *Fusarium*-damaged kernel contamination with quality indicators in common wheat and the agrometeorological parameters by region in Romania in the extremely dry 2023–2024 agricultural year.

**Pearson Correlation (r_{xy}) of Average *Fusarium*-damaged kernel (FDK) Contamination with the Average Quality Indicators in Common Wheat and the Average Agrometeorological Parameters by Agricultural Region in Romania in the Extremely Dry 2023–2024 Agricultural Year**									
**Variable**			**Transylvania**	**Southern Hilly Area**	**West Plain**	**Oltenia Plain**	**Moldavia**	**Southern Plain**	**Dobrogea**
**Quality Indicators in Common Wheat**	**Microbiological and Mycotoxicological**	**Moisture, M**	-0.091	-0.023	-0.031	-0.080	-0.388	0.352	N.a.
		**Water Activity, aw**	-0.057	-0.206	-0.401	0.024	-0.392	0.076	N.a.
		**Total Fungi**	0.052	0.034	0.073	0.669 ***	0.273	-0.097	N.a.
		**Deoxynivalenol, DON**	0.939 ***	0.619 **	0.911 ***	0.717 ***	0.966 ***	0.689 ***	N.a.
		**Total Aflatoxin, AF**	0.181	0.003	0.528 *	-0.175	0.002	-0.366	N.a.
	**Physico-chemical**	**Hectolitre Mass, HM**	-0.208	-0.124	-0.580 **	-0.660 ***	-0.122	-0.253	N.a.
		**Hagberg Falling Number, HFN**	-0.145	-0.064	-0.059	0.857 ***	-0.178	-0.212	N.a.
		**Protein, P**	0.046	-0.151	-0.785 ***	-0.538 **	-0.591 **	-0.371	N.a.
		**Wet Gluten, WG**	0.067	0.007	-0746 ***	-0.518	-0.630 **	-0.341	N.a.
		**Wet Gluten Deformation Index, WGDI**	-0.100	-0.143	-0.250	-0.011	0.378	-0.367	N.a.
		**Gluten Index, GI**	-0.125	0.146	-0.693 ***	0.606 **	0.308	0.227	N.a.
	**Sensory–colorimetric**	**L*—whiteness**	-0.001	0.284	0.476 *	0.125	0.558 **	0.452 *	N.a.
		**a*—redness**	-0.172	-0.325	-0.358	-0.267	-0.442 *	-0.495 *	N.a.
		**b*—yellowness**	-0.347	-0.289	-0.518 *	0.171	-0.135	-0.488 *	N.a.
**Agrometeorological Parameters**	**Air Temperature**	**May 2024**	0.198	0.427 *	-0.084	0.987 ***	-0.027	-0.230	N.a.
		**June 2024**	0.282	-0.183	-0.173	0.053	0.107	-0.626 **	N.a.
		**July 2024**	0.224	0.012	-0.181	0.394	0.147	-0.372	N.a.
		**August 2024**	0.166	0.273	-0.020	-0.871 ***	0.126	-0.566 *	N.a.
		**Annual, September 2023–August 2024**	0.168	0.285	0.032	0.980 ***	0.101	-0.502 *	N.a.
	**Precipitation**	**May 2024**	-0.092	0.783 ***	0.039	0.543	-0.181	-0.418	N.a.
		**June 2024**	-0.009	-0.184	0.578 **	-0.307	0.275	0.282	N.a.
		**July 2024**	-0.340	-0.281	-0.432 *	-0.963 ***	-0.367	0.101	N.a.
		**August 2024**	0.121	0.232	0.539 **	0.481 *	0.025	0.016	N.a.
		**Annual, September 2023–August 2024**	0.002	0.316	0.566 **	-0.172	-0.128	0.056	N.a.
	**Soil Water Reserve**	**September 2023 (0–20 cm)**	-0.013	0.300	-0.062	0.287	-0.128	0.101	N.a.
		**October 2023 (0–20 cm)**	0.132	0.340	-0.069	0.360	-0.157	-0.379	N.a.
		**November 2023 (0–50 cm)**	0.152	0.454 *	0.045	0.303	0.220	-0.395	N.a.
		**March 2024 (0–100 cm)**	-0.020	0.005	0.423 *	0.292	-0.138	-0.480 *	N.a.
		**April 2024 (0–100 cm)**	-0.018	0.191	0.532 *	0.368	-0.195	-0.378	N.a.
		**May 2024 (0–100 cm)**	0.070	0.568 **	0.545 **	0.478 *	-0.036	-0.474 *	N.a.
		**June 2024 (0–100 cm)**	0.046	0.646 **	0.431 *	0.393	0.167	-0.565 **	N.a.
		**Annual, September 2023–June 2024**	0.020	0.492 *	0.324	0.526 *	-0.006	-0.558 **	N.a.

Significance (two-tailed): * *p*-value < 0.05 – significant differences, ** *p*-value < 0.01 – distinct significant differences, *** *p*-value < 0.001 – very significant differences.

Table S5. Pearson correlation of deoxynivalenol contamination with quality indicators in common wheat and the agrometeorological parameters by region in Romania in the extremely dry 2023–2024 agricultural year.

Pearson Correlation (r_{xy}) of Average Deoxynivalenol (DON) Contamination with the Average Quality Indicators in Common Wheat and the Average Agrometeorological Parameters by Agricultural Region in Romania in the Extremely Dry 2023–2024 Agricultural Year

Variable			Transylvania	Southern Hilly Area	West Plain	Oltenia Plain	Moldavia	Southern Plain	Dobrogea
Quality Indicators in Common Wheat	Microbiological and Mycotoxicological	Moisture, M	-0.086	0.115	-0.120	-0.073	-0.286	-0.596 **	-0.404
		Water Activity, aw	-0.038	0.041	-0.306	-0.059	-0.303	-0.341	-0.965 ***
		Total Fungi	0.077	0.201	-0.003	0.612 **	0.252	-0.175	-0.196
		Fusarium-damaged kernel, FDK	0.939 ***	0.619 **	0.911 ***	0.717 ***	0.966 ***	0.689 ***	NaN
		Total Aflatoxin, AF	0.194	0.185	0.535 *	-0.256	0.084	-0.144	0.267
	Physico-chemical	Hectolitre Mass, HM	-0.255	-0.323	-0.504 *	-0.772 ***	-0.117	0.145	0.777 ***
		Hagberg Falling Number, HFN	-0.148	-0.273	-0.137	0.778 ***	-0.183	0.173	0.435 *
		Protein, P	-0.029	-0.347	0.699 ***	-0.264	-0.520 *	0.005	-0.008
		Wet Gluten, WG	0.023	-0.353	-0.723 ***	-0.111	-0.532 *	0.024	-0.071
		Wet Gluten Deformation Index, WGDI	-0.118	-0.101	-0.027	0.148	-0.334	-0.242	-0.321
		Gluten Index, GI	-0.162	0.121	-0.687 ***	0.327	0.246	0.076	0.216
	Sensory–colorimetric	L*—whiteness	0.128	0.210	0.607 **	-0.163	0.492 *	0.121	0.694 ***
		a*—redness	-0.280	-0.231	-0.546 *	-0.001	-0.385	0.025	0.494 *
		b*—yellowness	-0.358	-0.205	-0.686 ***	0.363	-0.103	0.128	0.491 *
Agrometeorological Parameters	Air Temperature	May 2024	0.208	0.108	-0.241	0.788 ***	-0.014	-0.446 *	0.318
		June 2024	0.322	0.038	-0.467 *	-0.020	0.129	-0.231	0.318
		July 2024	0.235	0.132	-0.463 *	0.260	0.174	-0.277	0.318
		August 2024	0.173	0.291	-0.348	-0.725 ***	0.149	-0.307	0.319
		Annual, September 2023–August 2024	0.181	-0.027	-0.241	-0.800 ***	0.124	-0.335	0.317
	Precipitation	May 2024	-0.021	0.717 ***	-0.255	0.493 *	-0.172	-0.476 *	-0.315
		June 2024	-0.022	-0.545 **	0.760 ***	-0.185	0.238	-0.059	0.326
		July 2024	-0.341	-0.797 ***	-0.424	-0.761 ***	-0.387	-0.142	0.326
		August 2024	0.124	0.307	0.513 *	0.359	0.081	0.095	0.317
		Annual, September 2023–August 2024	-0.003	-0.103	0.790 ***	-0.075	-0.130	-0.265	-0.318
	Soil Water Reserve	September 2023 (0–20 cm)	-0.007	0.517 *	0.296	0.252	-0.112	0.185	0.318
		October 2023 (0–20 cm)	0.153	0.633 **	0.291	0.317	-0.169	0.026	0.318
		November 2023 (0–50 cm)	0.114	0.620 **	0.369	0.266	0.210	-0.352	0.318
		March 2024 (0–100 cm)	-0.037	0.353	0.701 ***	0.257	-0.093	-0.578 **	0.318
		April 2024 (0–100 cm)	-0.039	0.485 *	0.770 ***	0.324	-0.167	-0.098	0.318
		May 2024 (0–100 cm)	0.080	0.672 ***	0.761 ***	0.423 *	-0.017	-0.286	0.318
		June 2024 (0–100 cm)	0.038	0.736 ***	0.710 ***	0.347	-0.151	-0.541 **	0.318
		Annual, September 2023–June 2024	0.004	0.666 ***	0.586 **	0.256	0.223	-0.368	0.318

Significance (two-tailed): * p-value < 0.05 – significant differences, ** p-value < 0.01 – distinct significant differences, *** p-value < 0.001 – very significant differences.

Table S6. Pearson correlation of total aflatoxin contamination with quality indicators in common wheat and the agrometeorological conditions by region in Romania in the extremely dry 2023–2024 agricultural year.

Pearson Correlation (r_{xy}) of Average Total Aflatoxin (AF) Contamination with the Average Quality Indicators in Common Wheat and the Average Agrometeorological Parameters by Agricultural Region in Romania in the Extremely Dry 2023–2024 Agricultural Year									
Variable			Transylvania	Southern Hilly Area	West Plain	Oltenia Plain	Moldavia	Southern Plain	Dobrogea
Quality Indicators in Common Wheat	Microbiological and Mycotoxicological	Moisture, M	-0.159	0.056	-0.530 *	0.106	0.165	-0.212	-0.420
		Water Activity, aw	-0.019	-0.228	0.064	0.029	0.065	-0.249	-0.320
		Total Fungi	-0.163	0.114	0.058	0.035	0.090	-0.263	0.207
		Fusarium-damaged kernel, FDK	0.181	0.003	0.528 *	-0.175	0.002	-0.366	NaN
		Deoxynivalenol, DON	0.194	0.185	0.535 *	-0.256	0.084	-0.144	0.267
	Physico-chemical	Hectolitre Mass, HM	-0.467 *	-0.212	-0.254	0.129	-0.071	-0.133	-0.011
		Hagberg Falling Number, HFN	-0.412	-0.060	-0.039	-0.178	0.113	-0.077	0.387
		Protein, P	-0.046	-0.263	-0.336	-0.053	-0.115	0.073	0.361
		Wet Gluten, WG	0.019	-0.118	-0.162	-0.085	-0.075	0.140	0.335
		Wet Gluten Deformation Index, WGDI	-0.256	0.022	-0.012	-0.124	-0.122	0.108	-0.094
		Gluten Index, GI	0.076	-0.062	-0.213	-0.113	-0.299	-0.255	-0.251
	Sensory–colorimetric	L*—whiteness	0.172	0.136	0.109	0.192	-0.304	-0.407	0.385
		a*—redness	-0.137	-0.020	-0.068	-0.104	0.097	0.346	0.268
		b*—yellowness	-0.322	0.016	-0.187	-0.180	-0.063	0.240	0.359
Agrometeorological Parameters	Air Temperature	May 2024	0.288	0.093	0.334	-0.187	-0.005	-0.104	-0.239
		June 2024	0.024	0.188	-0.087	-0.128	-0.125	0.141	-0.239
		July 2024	0.217	0.134	-0.054	-0.179	-0.112	0.095	-0.239
		August 2024	0.253	0.294	-0.061	0.111	-0.052	0.084	-0.239
		Annual, September 2023–August 2024	0.241	0.074	0.174	-0.156	-0.086	0.066	-0.239
	Precipitation	May 2024	-0.362	0.016	-0.606 **	-0.002	0.238	-0.147	0.235
		June 2024	0.003	0.150	0.322	-0.169	0.303	-0.243	-0.249
		July 2024	-0.234	-0.188	0.205	0.206	-0.139	-0.246	-0.229
		August 2024	-0.323	0.004	-0.085	-0.209	-0.254	-0.150	0.242
		Annual, September 2023–August 2024	0.123	-0.022	-0.578 **	0.148	0.095	-0.328	0.239
	Soil Water Reserve	September 2023 (0–20 cm)	-0.105	0.061	0.361	-0.053	0.417	0.236	0.239
		October 2023 (0–20 cm)	-0.090	0.099	0.331	-0.057	0.272	0.196	0.239
		November 2023 (0–50 cm)	0.127	0.178	0.547 **	-0.054	0.054	-0.066	0.239
		March 2024 (0–100 cm)	0.061	0.032	0.583 **	-0.054	-0.126	-0.247	0.239
		April 2024 (0–100 cm)	0.054	-0.028	0.472 *	-0.058	0.109	-0.175	0.239
		May 2024 (0–100 cm)	-0.031	-0.108	0.377	-0.630	0.513 *	-0.194	0.239
		June 2024 (0–100 cm)	-0.087	0.227	0.535 *	-0.059	0.310	-0.219	0.239
		Annual, September 2023–June 2024	0.048	-0.139	0.483 *	0.009	0.239	-0.173	0.239

Significance (two-tailed): * *p*-value < 0.05 – Significant Differences, ** *p*-value < 0.01 – Distinct Significant Differences, *** *p*-value < 0.001 – Very Significant Differences.
